# Medical Aid and Nursing for the Poor

**Published:** 1908-07

**Authors:** 


### MEDICAL AID AND NURSING FOR THE POOR.

---

A worthy movement has recently been inaugurated in Atlanta to establish a dispensary district Nursing Association, which is to have as its aim the promotion of medical and educational work among the poor.

This work is headed by the Federation of Woman's Clubs. The plan to carry out this work is as follows:

"First, by establishing dispensaries and clinics, and doing district nursing by Christian nurses.

"Second, by co-operating with the municipal authorities in endeavoring to improve the city's care of the poor, outside the hospitals.

"Third, by co-operating with other charitable organizations for the care of the sick poor.

"Fourth, by doing such educational work as lectures, demonstrations, distributing tracts, and organizing clubs for boys and girls."

